# ASSESSING PREFERENCES FOR COMMUNICATING WITH TECHNOLOGY: A PERSON-CENTERED APPROACH TO CARE MANAGEMENT

**DOI:** 10.1093/geroni/igac059.608

**Published:** 2022-12-20

**Authors:** Andrea Sillner, Marie Boltz, Logan Sweeder, Kimberly Van Haitsma

**Affiliations:** Pennsylvania State University Ross and Carol Nese College of Nursing, University Park, Pennsylvania, United States; Penn State, Pennsylvania State University, Pennsylvania, United States; The Pennsylvania State University, State College, Pennsylvania, United States; Pennsylvania State University, University Park, Pennsylvania, United States

## Abstract

Technological advances, such as telehealth, have been used to manage the multiple chronic conditions that impact over 25% of the US adult population. Technology-assisted communication (TAC) can help to bridge the gap in effective management of health conditions in the community by patients, informal caregivers, and healthcare providers, while emphasizing person-centered care. The purpose of this project was to develop a new theoretically-derived and evidence based subscale for the Preferences for Everyday Living Inventory (PELI) that addresses preferred TAC approaches for community-dwelling adults over the age of 50 years in the context of multiple chronic conditions (N=297). Results indicated that over 60% of older adults are satisfied with technology-based healthcare communications. In general, older adults in the sample are satisfied with all domains of technology-assisted communication that are asked within P-TAC, including timing, sending and receiving of information, and content of communications. Almost 80% (N=234) indicate that they are satisfied with the content of TAC. This research has lead to the development of assessment items that will allow providers to better assess and then integrate patient preferences for technology communication strategies into plans of care. Potential benefits of understanding preferences for TAC include alignment of chronic care management with preferred strategies which may lead to improvement care congruence and improved healthcare outcomes for the older adult.

